# Role of drug transporters in the sensitivity of acute myeloid leukemia to sorafenib

**DOI:** 10.18632/oncotarget.25494

**Published:** 2018-06-19

**Authors:** Rocio I.R. Macias, Anabel Sánchez-Martín, Gabriela Rodríguez-Macías, Luis I. Sánchez-Abarca, Elisa Lozano, Elisa Herraez, Maria D. Odero, José L. Díez-Martín, Jose J.G. Marin, Oscar Briz

**Affiliations:** ^1^ Laboratory of Experimental Hepatology and Drug Targeting (HEVEFARM), University of Salamanca, IBSAL, Salamanca, Spain; ^2^ National Institute for the Study of Liver and Gastrointestinal Diseases (CIBERehd), Madrid, Spain; ^3^ Department of Hematology-BMT Unit, Hospital General Universitario Gregorio Marañón, Madrid, Spain; ^4^ Department of Hematology, University Hospital of Salamanca, IBSAL, Salamanca, Spain; ^5^ Department of Biochemistry and Genetics and CIMA, University of Navarra, Pamplona, CIBERONC, Spain; ^6^ Gregorio Marañon Institute for Health Research (IISGM), Madrid, Spain

**Keywords:** AML, cancer, chemoresistance, chemotherapy, tyrosine kinase inhibitor

## Abstract

**Background:**

Chemoresistance often limits the success of the pharmacological treatment in acute myeloid leukemia (AML) patients. Although positive results have been obtained with tyrosine kinase inhibitors (TKIs), such as sorafenib, especially in patients with Fms-like tyrosine kinase 3 (FLT3)-positive AML, the success of chemotherapy is very heterogeneous. Here we have investigated *in vitro* whether the transportome (set of expressed plasma membrane transporters) is involved in the differential response of AML to sorafenib.

**Methods:**

The sensitivity to sorafenib-induced cell death (MTT test and anexin V/7-AAD method) was evaluated in five different cell lines: MOLM-13, OCI-AML2, HL-60, HEL and K-562. The transportome was characterized by measuring mRNA using RT-qPCR. Drug uptake/efflux was determined by flow cytometry using specific substrates and inhibitors.

**Results:**

The cytostatic response to sorafenib was: MOLM-13>>OCI-AML2>HL-60>HEL≈K-562. Regarding efflux pumps, MDR1 was highly expressed in HEL>K-562≈MOLM-13, but not in OCI-AML2 and HL-60. BCRP and MPR3 expression was low in all cell lines, whereas MRP4 and MRP5 expression was from moderate to high. Flow cytometry studies demonstrated that MRP4, but not MRP5, was functional. The expression of the organic cation transporter 1 (OCT1), involved in sorafenib uptake, was MOLM-13>OCI-AML2≈HL-60 and non detectable in HEL and K-562. Transfection of HEL cells with OCT1 increased the sensitivity of these cells to sorafenib, whereas inactive genetic variants failed to induce this change.

**Conclusion:**

Together with changes in the expression/function of receptors targeted by TKIs, the expression of plasma membrane transporters involved in sorafenib uptake/efflux may affect the response of leukemia cells to this drug.

## INTRODUCTION

Acute myeloid leukemia (AML) is a hematological neoplasm characterized by abnormal differentiation and increased proliferation of myeloid precursor cells, which accumulate in bone marrow, blood and tissues. A major problem in the treatment of this malignancy is that two-thirds of patients fail to respond to available chemotherapy and, in some cases, after an initial promising response, there is a relapse due to the development of drug resistance, which contributes to the poor long-term survival of many patients suffering from AML. Significant efforts are being conducted to develop more effective treatments as well as to identify biomarkers able to predict the lack of response of this cancer to chemotherapy.

Sorafenib is a targeted drug that has been approved in the USA and Europe for treating renal cell carcinoma, hepatocellular carcinoma and thyroid carcinoma [1–3]. This organic compound belongs to the family of tyrosine kinase inhibitors (TKIs) able to inhibit several plasma membrane receptors (TKRs) whose tyrosine kinase activity is crucial for their participation in multiple pathways involved in the evolution and progression of these malignancies.

Thus, sorafenib is able to interact with Fms-like tyrosine kinase 3 (FLT3) receptor, together with RAS/RAF, members of the platelet-derived growth factor (PDGF) receptor family (PDGFR and Kit) and vascular endothelial growth factor (VEGF) receptors 2 and 3 [4]. Of particular interest are the activating mutations in the *FLT3* gene, such as internal tandem duplication (FLT3-ITD) or D835-activating point mutations (FLT3-TKD), which lead to the overexpression or constitutive activation of the kinase, and occur in about 30% of AML cases [5]. The presence of the *FLT3/ITD* mutation is associated with poor prognosis in AML [6, 7], but several preclinical studies and clinical trials support the concept that sorafenib could be effective for the treatment of patients with AML, especially of those harboring *FTL3* mutations. Promising beneficial response rate has been obtained using this drug either administered alone, in combination with other chemotherapeutic agents, or as maintenance chemotherapy after allogeneic stem cell transplantation [8–14].

Unfortunately, chemoresistance constitutes a major limitation for the successful treatment of AML with sorafenib. The mechanisms accounting for the lack of response to this drug are not completely understood. The transportome, defined as the plasma membrane transporters expressed at any given moment, is involved in the uptake and efflux of drugs, and hence accounts for the amount of active drug that reaches its intracellular targets (for a review see [15]). The organic cation transporter-1 (OCT1, gene *symbol SLC22A1*) plays a major role in the uptake of sorafenib [16] and, at least in solid tumors, a decrease in the expression of this transporter and the presence of inactivating mutations are frequent [16]. Among other mechanisms of chemoresistance, changes in the transportome, such as overexpression of several export pumps, belonging to the superfamily of ATP binding cassette (ABC) proteins, and down-regulation of uptake transporters together with the presence of polymorphisms with loss of function, have been associated with the reduced sensitivity to sorafenib in different types of cancer [17–20].

The aim of the present study was to investigate the role of the transportome of AML lines in their differential sensitivity to sorafenib.

## RESULTS

### Sensitivity of leukemic cells to sorafenib

**Figure 1:**
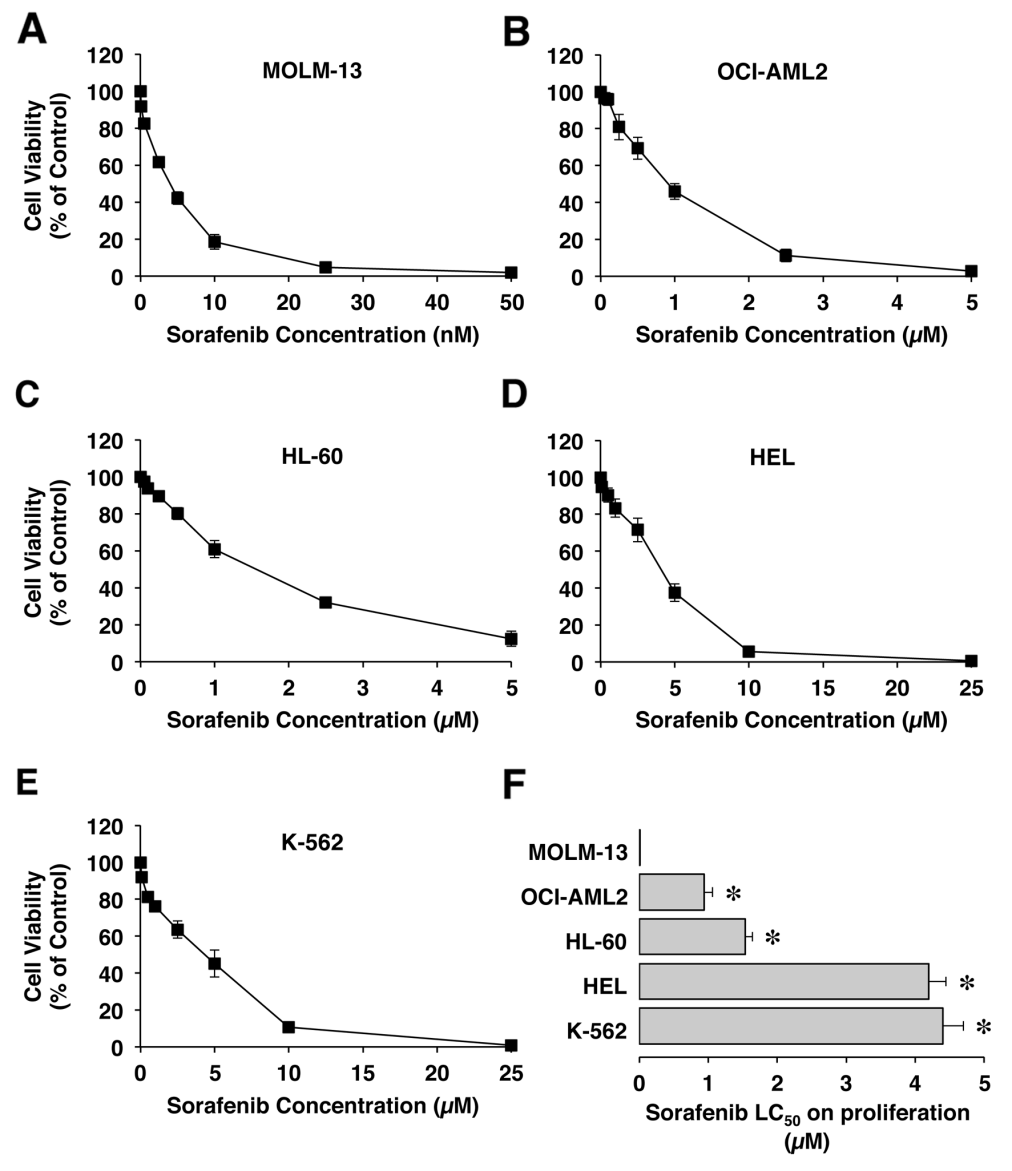
Concentration-dependent effect of sorafenib on cell viability determined with the MTT-formazan test after exposure of MOLM-13 **(A)**, OCI-AML2 **(B)**, HL-60 **(C)**, HEL **(D)** and K-562 **(E)** cells to this drug for 72 h. Values are expressed as percentages of controls (cells incubated in the absence of drug). Comparison of mean LC_50_ in cell lines **(F)**. Values (means±SEM) are expressed as percentages of controls (cells incubated in the absence of drug) from 4 experiments performed in triplicate. ^*^, p<0.05, as compared with MOLM-13 cell line by the Bonferroni method of multiple comparison test.

The cytostatic effect of sorafenib was determined in a panel of five human cell lines; four AML -OCI-AML2, HL-60, MOLM-13 and HEL- and one derived from chronic myeloid leukemia (K-562). Information regarding subtypes of AML classification according to the French-American-British (FAB) cooperative group’s criteria and the World Health Organization (WHO) and typical mutations is shown in Table 1. Sorafenib induced a dose dependent reduction in cell viability in all these cell lines (Figures 1A-1E). The sensitivity to sorafenib was markedly higher for MOLM-13 cells than for the rest of cells tested. The comparison of the concentrations able to induce lethal effect on 50% of these cells (LC_50_) (Figure 1F) revealed that LC_50_ was in the μM range for most cell lines, except for MOLM-13, which was in the nM range.

**Table 1:** Characteristics of acute myeloid leukemia (AML) and K-562 cell lines

Cell line	FAB	WHO		AML	Mutations
**MOLM-13**	M5	monocytic leukemia		sAML	*FLT3 ITD*
**OCI-AML2**	M4	monocytic leukemia		*de novo*	*DNMT3A*
**HL-60**	M2	AML with maturation		*de novo*	
**HEL**	M6	erythroid leukemia		sAML	*JAK2*
**K-562**	*chronic myelogenous leukemia*				

Abbreviations: FAB, French-American-British classification system; sAML, secondary AML; WHO, World Health Organization classification system.

**Figure 2:**
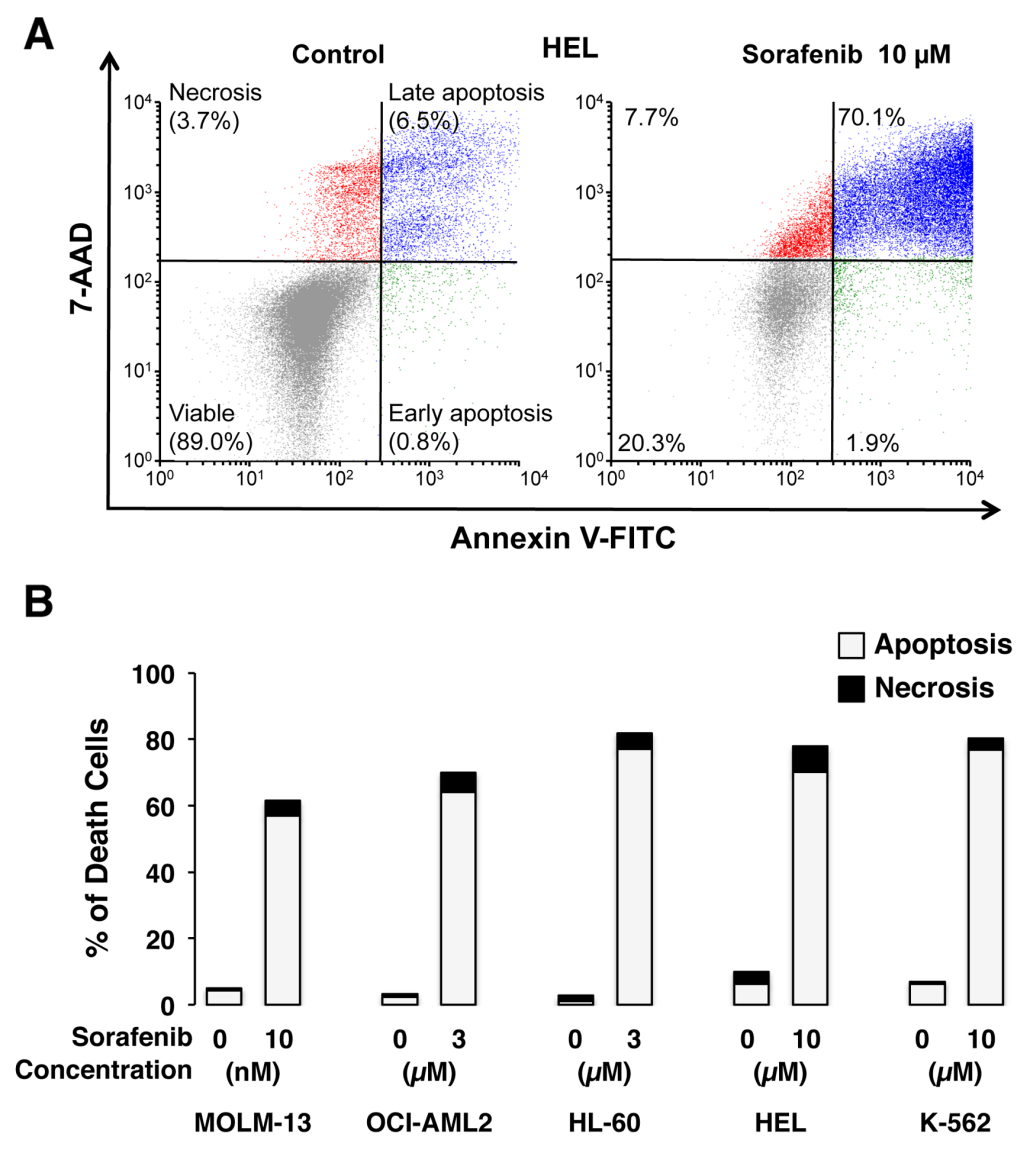
Representative images of HEL cells untreated (Control) and exposed to 10 μM sorafenib After 72 h cells were incubated with Annexin V-PE in a buffer containing 7-amino-actinomycin (7-AAD) and analyzed by flow cytometry **(A)**. A large population of cells was annexin V-PE and 7-AAD positive, indicating that they were in late stage apoptosis. Percentage of cell death due to apoptosis/necrosis in myeloid leukemia cell lines MOLM-13, OCI-AML2, HL-60, HEL and K-562 exposed to the indicated concentrations of sorafenib for 72 h analyzed by flow cytometry after incubation with anexin V-PE and 7-AAD **(B)**. Values are expressed as the mean of three experiments performed in duplicate and SEM was always <10%.

Sorafenib-induced cell death was determined by flow cytometry. As a representative analysis, results obtained with HEL cells are shown in Figure 2A. Similar assays were carried out with the rest of the cell lines analyzed here (data not shown). Thus, concentration-dependent studies indicated that sorafenib-induced cell death was mainly due to the enhanced activation of apoptosis with a minor contribution of necrosis in all cell lines at all concentrations assayed (Figure 2B).

### Characterization of basal sorafenib-related transportome in leukemic cells

**Figure 3:**
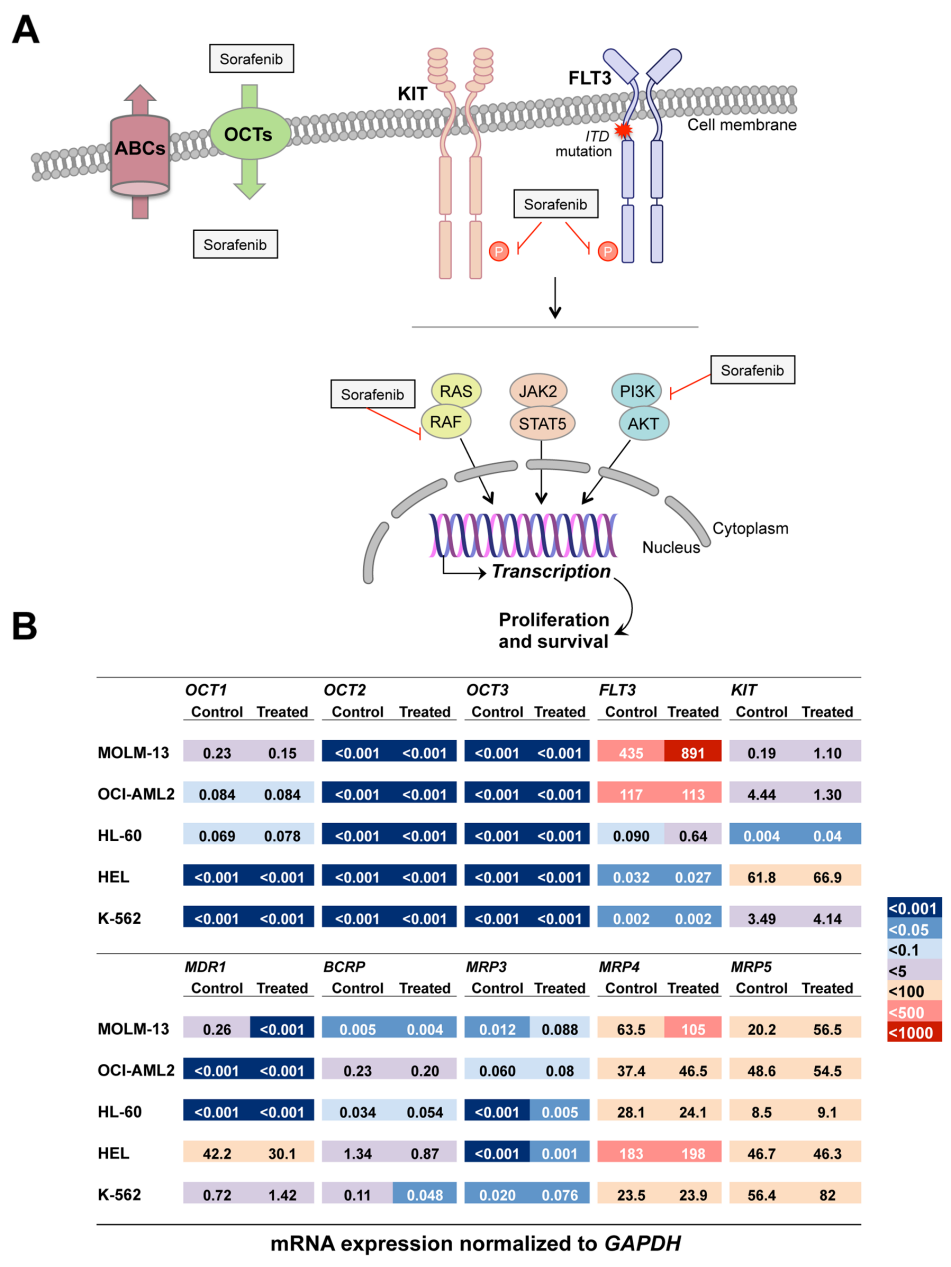
Schematic representation of sorafenib uptake transporters (organic cation transporters, OCTs), export pumps (ATP-binding cassette proteins, ABCs) and targets present in myeloid leukemia cells **(A)**. Basal and sorafenib-induced mRNA expression normalized to GAPDH of transporters and targets of sorafenib in MOLM-13, OCI-AML2, HL-60, HEL and K-562 cells **(B)** determined by RT-qPCR.

Basal levels of mRNA for some genes involved in the uptake (OCTs), the export (ABCs) and the response (TKRs) to sorafenib (see scheme in Figure 3A) were determined. Levels of *SLC22A2* and *SLC22A3* mRNA, encoding for OCT2 and OCT3, respectively, were undetectable in all cell lines. The expression of OCT1 was evident in MOLM-13. The levels of *SLC22A1* mRNA were three-fold higher in MOLM-13 than in OCI-AML2 and HL-60 cells, but were undetectable in the HEL and K-562 cells (Figure 3B).

Regarding export pumps, the multidrug resistance protein (MDR1, gene symbol *ABCB1*) was not detectable in OCI-AML2 and HL-60 cells, but was highly expressed in HEL>>K-562>MOLM-13 cells. Expression levels of the breast cancer resistance protein (BCRP, gene symbol *ABCG2*) were HEL>OCI-AML2>K-562>HL-60>MOLM-13 cells. Among members of the multidrug resistance-associated proteins (MRPs) family, several potential candidates for being involved in sorafenib chemoresistance were investigated. MRP2 (gene symbol *ABCC2*) was not detectable in any cell line (data not shown). MRP3 (gene symbol *ABCC3*) was not expressed in HL-60 and HEL cells, but *ABCC3* mRNA was detected at low levels in OCI-AML2, MOLM-13 and K-562. In contrast, both MRP4 (gene symbol *ABCC4*) and MRP5 (gene symbol *ABCC5*) were highly expressed in all five cell lines.

To investigate the relationship between the response to sorafenib and the expression of its targets, *FLT3* and *KIT* mRNA levels were also determined (Figure 3B). In MOLM-13 cells, *FLT3* expression was 4-fold higher than in OCI-AML2 and >4,000-fold higher than in the rest of the cells, where *FLT3* expression was negligible. Regarding *KIT* expression, this was HEL>OCI-AML2≈K-562>MOLM-13>>HL-60.

### Treatment-induced changes in sorafenib-related transportome in leukemic cells

To evaluate whether the expression profile of transport proteins was modified in response to the exposure of leukemia cells to sorafenib, the cells were incubated with this drug at the LC_50_ of each cell line for 72 h. This maneuver resulted in an enhanced expression of several genes and the down-regulation of others (Figure 3B). OCT1 expression was not affected. In contrast, significant changes in the expression of export pumps associated with chemoresistance were observed, mainly in MOLM-13 cells, where treatment with sorafenib induced the up-regulation of MRP4 and MRP5. Regarding sorafenib molecular targets, an increase in *FLT3* expression was observed in MOLM-13 and HL-60 cells, while *KIT* expression was enhanced in MOLM-13 and reduced in OCI-AML2 cells.

### Functional studies in leukemic cells

**Figure 4:**
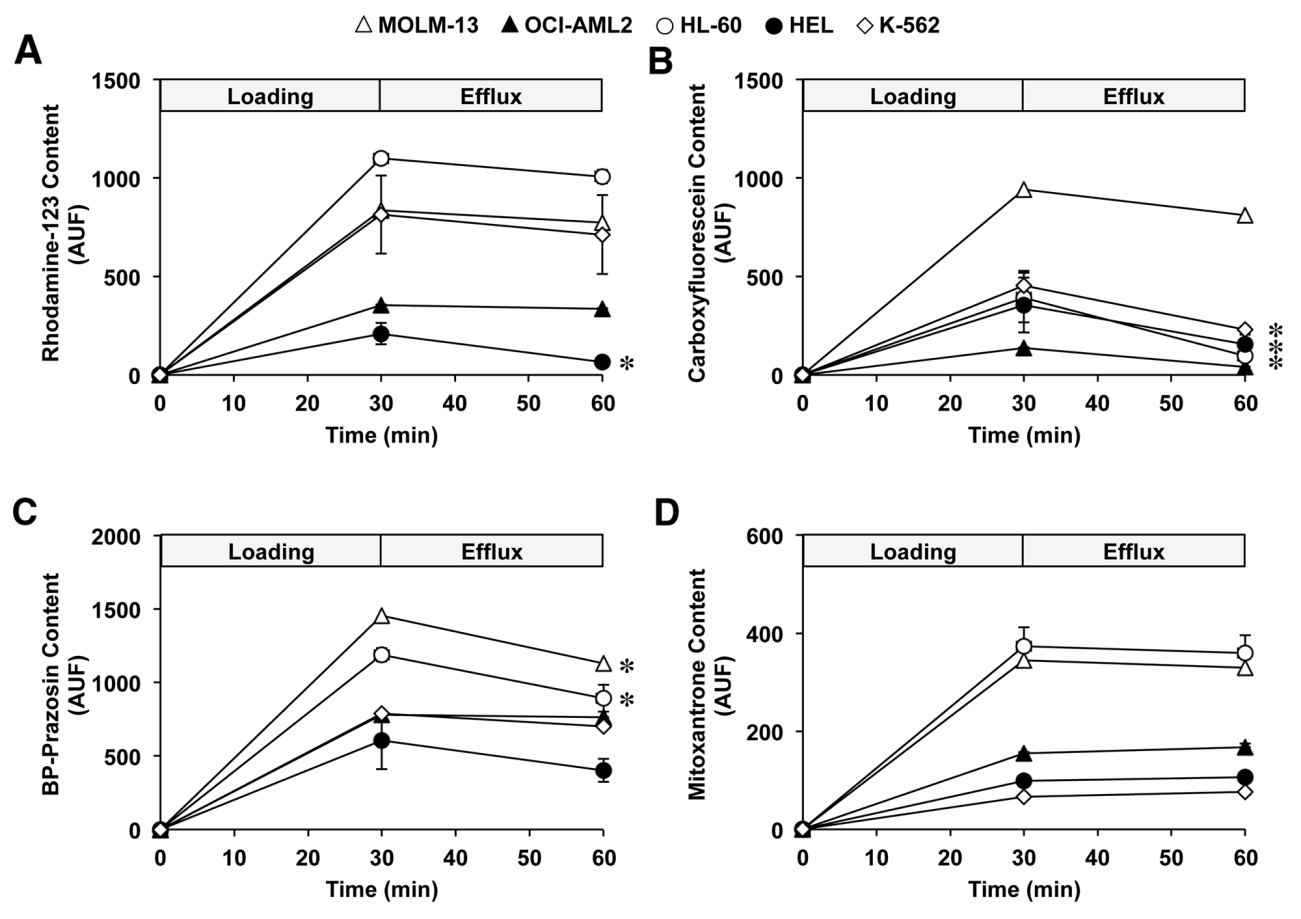
Time course of cell content of fluorescent ABC substrates in myeloid leukemia cells MOLM-13, OCI-AML2, HL-60, HEL and K-562 during loading and efflux periods After the cells were loaded with 1 μM rhodamine-123 **(A)**, 1 μM carboxyfluorescein diacetate **(B)**, 1 μM BODIPY-prazosin **(C)** or 25 μM mitoxantrone **(D)** at 37°C for 30 min (loading period) they were diluted 1:10 with substrate-free medium and incubated at 37°C for 30 min. Values (means±SEM) were determined by flow cytometry from 3 different cultures. ^*^, p<0.05, as compared to the substrate content after loading period.

**Figure 5:**
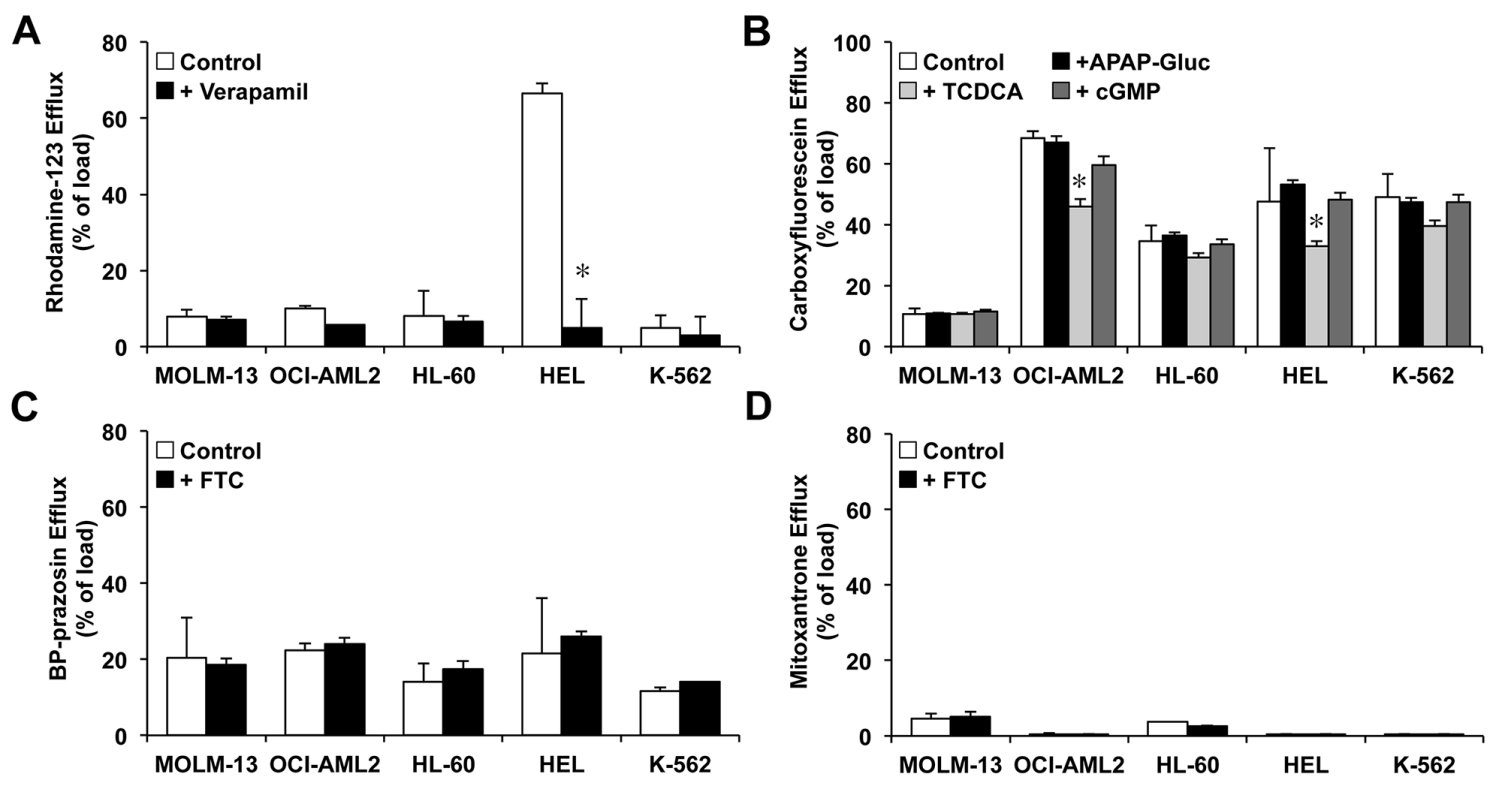
Efflux of fluorescent ABC substrates from preloaded myeloid leukemia cells MOLM-13, OCI-AML2, HL-60, HEL and K-562, which reflects changes in the mean fluorescence as compared with that found at the end of the loading period (Figure 4) After the cells were loaded with 1 μM rhodamine-123 **(A)**, 1 μM carboxyfluorescein diacetate **(B)**, 1 μM BODIPY-prazosin **(C)** or 25 μM mitroxantrone **(D)** at 37°C for 30 min (loading period) they were diluted 1:10 with substrate-free medium containing, or not, the specific ABC inhibitors—10 μM verapamil (MDR1), 100 μM APAP glucuronide (MRP3), 100 μM taurochenodeoxycholic acid (TCDCA) (MRP3 and MRP4), 500 μM cGMP (MRP4 and MRP5) and 5 μM fumitremorgin C (BCRP)—and incubated at 37°C for 30 min. Values (means±SEM) were determined by flow cytometry from 3 different cultures. ^*^, p<0.05, as compared to the substrate content after loading period.

The functionality of transporters involved in determining the content of sorafenib in leukemic cells revealed that, in general, the results were consistent with data regarding the expression of ABC pumps. High activity of MDR1 was only observed in HEL cells, as demonstrated by the low rhodamine-123 intracellular content after 30 min (Figure 4A) and the significant reduction of the efflux of rhodamine-123 in presence of verapamil (Figure 5A).

Cell load of carboxyfluorescein during the uptake period was lower in OCI-AML2, HL-60, HEL and K-562 than in MOL-13 cells (Figure 4B), suggesting that MRP3-5 activity was lower in MOLM-13 cells. No reduction of the efflux of the substrate was found in the presence of MRP3 and MRP4/5 inhibitors, p-acetamidophenyl β-D-glucuronide (APAP-Gluc) and guanosine 3′,5′-cyclic monophosphate (cGMP), respectively (Figure 5B). These results suggested low activity of these pumps in all cell lines assayed. A significant reduction in carboxyfluorescein efflux by OCI-AML2 and HEL cells was observed in the presence of taurochenodeoxycholic acid (TCDCA) (Figure 5B), which suggested that MRP4 was functional in these cell lines. Low activity of BCRP was found in all cell lines using two different substrates: BODIPY-prazosin (Figure 4C) and mitoxantrone (Figure 4D). In agreement with these results no detectable effect of the BCRP specific inhibitor fumitremorgin C was found (Figure 5C and 5D).

**Figure 6:**
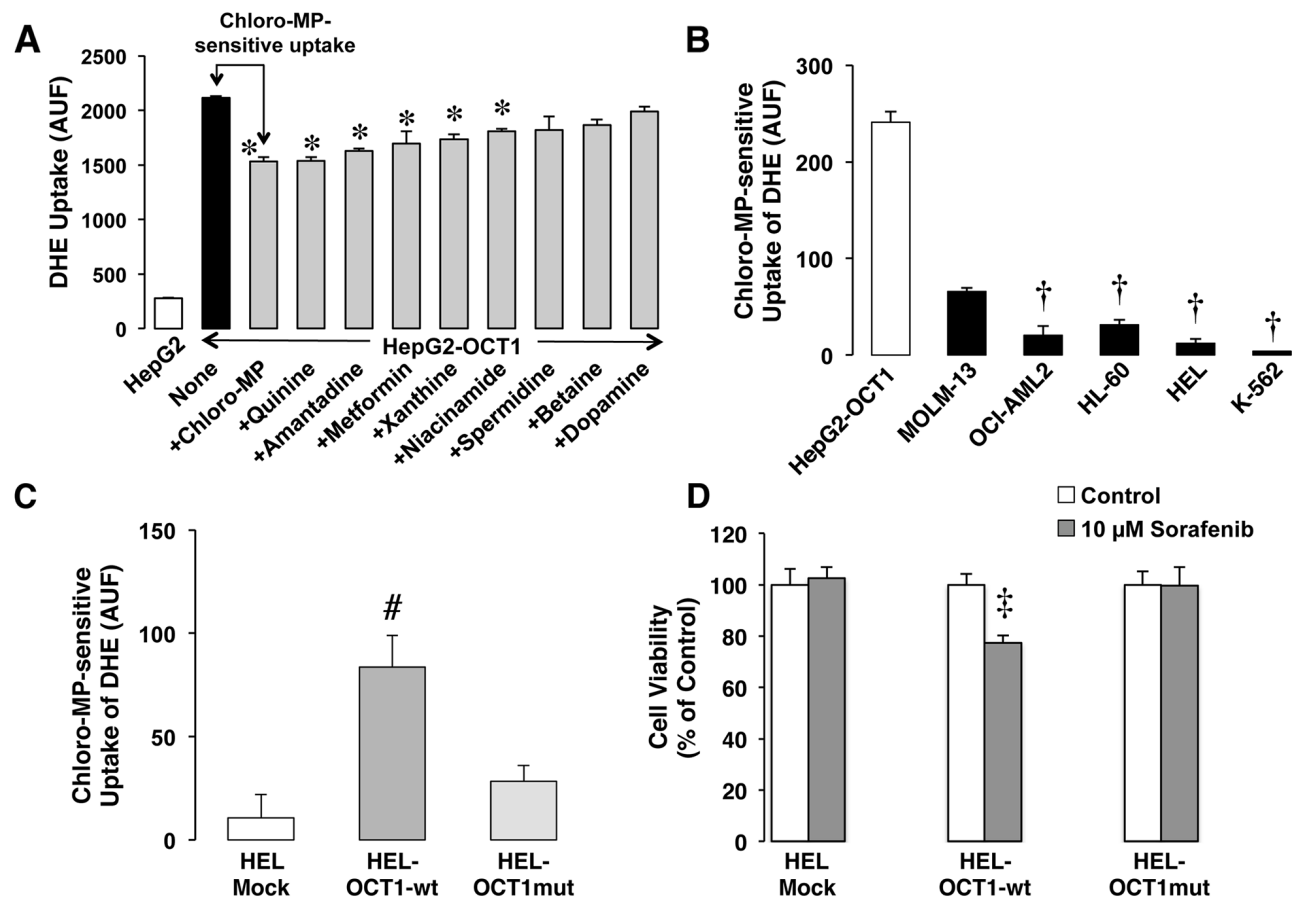
Dihydroethidium (DHE) uptake in mock HepG2 cells or stably expressing wild-type OCT1 in absence of inhibitors or in presence of one of the following compounds: chloro-MP, quinine, amantadine, metformine, xanthine, niacinamide, spermidine, betaine, dopamine **(A)**. Chloro-MP-sensitive uptake of DHE determined by flow cytometry **(B)**. The cells were incubated with uptake medium containing 5 μM DHE in the presence or absence of 100 μM chloro-MP for 30 min at 37°C before measuring fluorescence of cells. HepG2 stably expressing OCT1 were included as positive control. Effect of the expression of wild-type OCT1 or a mutated inactive variant on the chloro MP-sensitive uptake of DHE in HEL cells **(C)**. OCT1-induced sensitivity to sorafenib **(D)**. Effect of the expression of wild-type OCT1 or a mutated inactive variant on the viability of transfected HEL cells determined 72 hours after being incubated with 10 μM sorafenib for only 4 hours. Values are mean±SEM from three experiments performed in triplicate. The experiments were also carried out in non-transfected cells (Mock). Values are mean±SEM from two experiments performed in triplicate. ^*^, p<0.05, as compared with the HepG2 cells stably expressing OCT1; ^†^, p<0.05, as compared with MOLM-13 cells; ^#^, p<0.05, as compared with non-transfected cells (Mock); ^‡^, p<0.05, as compared with cells not treated with sorafenib (Control).

Finally, the functionality of OCT1 was studied by analyzing the ability of the cells to take up dihydroethidium (DHE) in an inhibitable manner. Liver HepG2 cells stably expressing OCT1 were used to test a panel of potential OCT1 inhibitors. The results showed that the strongest inhibitory effect was induced by 2-chloro-1-methylpyridinium iodide (chloro-MP) (Figure 6A). When chloro-MP-sensitive uptake of DHE was determined in all leukemia cells the following order was found: MOLM-13>HL-60≈OCI-AML2>HEL≈K-562 cells (Figure 6B).

### Sensitivity to sorafenib of HEL cells expressing OCT1

To further examine the contribution of OCT1 to the sensitivity of cells to sorafenib, HEL cells were selected because they have negligible basal expression of this transporter and the lowest sensitivity to sorafenib. HEL cells were transfected with either the wild-type or a mutated form of *OCT1* ORF. Expression of the former, but not the latter, conferred HEL cells the ability to take up DHE (Figure 6C). Moreover, OCT1 expression significantly sensitized HEL cells exposed to 10 μM sorafenib for a short period (4 h) as compared to cells either transfected with an empty vector (Mock) or the mutated non-functional form of OCT1 (Figure 6D).

## DISCUSSION

Some reports have suggested that AML cells with the *FLT3-ITD* mutation are more sensitive to sorafenib [8, 21]; however, a recent study has shown that *FLT3* wild-type cell lines can exhibit different sensitivities to this drug [22]. Also, it has been shown that the sensitivity of some *FLT3-ITD*^*+*^ cells to sorafenib can be rather poor [22]. In our study, MOLM-13 cells carrying the *FLT3-ITD* mutation were between 250-fold and 1000-fold more sensitive to sorafenib than the rest of cell lines assayed. Moreover, differences in the sensitivity to this drug among the cells without this mutation were found. Thus, other factors, in addition to the FLT3 status, can affect the response to this TKI. In this and other studies [22, 23], cell lines reflect the heterogeneity found in response to drugs, which is similar to the marked variability found in the drug response and survival outcomes of AML patients.

In order to reach its intracellular targets, sorafenib needs to enter the cells by crossing the plasma membrane. Owing to its ionic nature in aqueous solution, which means high polarity, this drug cannot diffuse across the plasma membrane. OCT1, which mediates the facilitated transport of structurally different organic cations, seems to play a major role in its uptake [16]. Moreover, the presence of ABC pumps able to export sorafenib can also affect the amount of active drug inside the cells [17, 18, 24]. Here we have investigated the relationship between the levels of uptake and efflux transporters that may affect the intracellular concentrations of sorafenib and the sensitivity of a panel of human myeloid leukemia cells.

Functional experiments using flow cytometry have also been carried out, since the quantification of mRNA levels may not always correlate with the amount of functional proteins due to the frequent presence of mutations that affect location and/or functionality. These studies demonstrated that, despite high levels of MRP5 in all the cell lines assayed here, this protein was not functional, because the MRP5 inhibitor cGMP did not affect the efflux of the MRP substrate carboxyfluorescein. High mRNA levels of MDR1 were only detected in HEL cells, and functional experiments confirmed that only this cell line was able to export rhodamine 123, a typical substrate of this pump. Moreover, the mRNA levels of BCRP were low in all the cell lines, and functional studies using fumitremorgin C as a specific inhibitor of the substrates BODIPY-prazosin and mitoxantrone confirmed a negligible functionality of this export pump.

The fact that the HEL cell line expresses low levels of OCT1 and high levels of MDR1 and MRP4 was consistent with its low sensitivity to sorafenib. Also K-562 cells presented extremely low levels of OCT1, a non-functional MDR1 and moderate MRP4 activity. However, the levels of molecular targets *FLT3* and *KIT* were lower in K-562 than in HEL cells, which could contribute to the poor sensitivity to sorafenib.

Regarding OCT1, only MOLM-13 and, to a lesser degree HL-60 and OCI-AML2 cells showed a significant OCT1-mediated uptake of sorafenib. The presence of *FLT3-ITD* mutation together with much higher expression levels of this molecular target in MOLM-13 could account for the marked sensitivity of this cell line. The fact that OCI-AML2 has higher levels of *FLT3* than the rest of cell lines *FLT3-ITD*^*-*^ can help to explain its superior sensitivity to sorafenib.

From these *in vitro* studies we can conclude that functional OCT1 expression is important in AML cell response to sorafenib. Low expression of OCT1 is frequent in solid tumors [16] and also in blasts of AML patients in comparison to healthy individuals [25]. These findings have clinical implications because the identification of the mechanisms involved in the chemoresistance to sorafenib can be useful to identify patients with scarce probability of responding to this treatment, and also to seek therapeutic strategies to enhance the efficacy of this chemotherapy. In support of this view it should be mentioned that, recently, the presence of OCT1 at the plasma membrane has been associated with better outcome of patients with advanced hepatocellular carcinoma treated with sorafenib [26]. The results from this *in vitro* study further support the need of carrying out clinical investigations to elucidate whether there is an association between the clinical response to sorafenib and the expression of functional transport proteins involved in sorafenib uptake and efflux in blasts of patients with AML.

## MATERIALS AND METHODS

### Chemicals

APAP-Gluc, carboxyfluorescein diacetate, cGMP, chloro-MP, DHE, fumitremorgin C, mitoxantrone, probenecid, rhodamine 123, taurochenodeoxycholic acid (TCDCA), thiazolyl blue tetrazolium bromide (MTT), and verapamil were obtained from Sigma-Aldrich Quimica (Madrid, Spain). BODIPY-prazosin was from Thermo Fisher and sorafenib tosylate from Santa Cruz Biotechnologies.

### Cell lines

Human cell lines HL-60, MOLM-13, HEL, OCI-AML2, derived from AML, and K-562 derived from chronic myelogenous leukemia in blast phase (CML-BP), were purchased from the DSMZ or ATCC cell line repositories and were grown in RPMI medium with 10% heat-inactivated fetal bovine serum (FBS), except OCI-AML2 cells in alpha-MEM medium and 20% FBS, supplemented with 100 U/ml penicillin and 100 μg/ml streptomycin, at 37°C and 5% CO_2_. All cell lines were routinely tested to ensure they were free of mycoplasm contamination (Mycoplasm Gel Form Kit, Biotools, Madrid, Spain).

### Cell viability assays

Cell viability was determined using MTT [27]. Briefly, cells were seeded in 96-well plates (5,000 or 10,000 cells per well) and exposed to increasing concentrations of sorafenib. Drug concentrations and controls (untreated cells) were set up 3-fold. After 72 h, MTT was added to each well and microplates maintained at 37°C with 5% CO_2_ for 4 h. The amount of MTT reduced to formazan is proportional to living cells. The purple formazan crystals were solubilized in 100 μl of 10% SDS in HCl 0.01 M and the absorbance measured in a microplate reader at 570 nm. The drug concentration required to reduce cell viability by 50% (LC_50_) was calculated from the dose-response curves.

Apoptosis was detected using the annexinV-PE/7 amino-actinomycin (7-AAD) apoptosis detection kit from BD Pharmigen. Cells were seeded in 48-well plates (250,000 cells per well) and exposed to increasing concentrations of sorafenib. After 72 h, cells were washed and resuspended in binding buffer (1:10 diluted in phosphate-buffered saline). Annexin V-PE (5 μl) and 7-AAD (5 μl) were added for 15 min. For every condition 50,000 events were collected and analyzed. Paint-A-Gate Pro software (BD) was used for the analyses.

OCT1-induced sensitivity to sorafenib was studied in HEL cells transiently transfected with plasmids containing the ORF of wild-type OCT1 or an inactive mutated form (c.181delCGinsT) [16] with Lipofectamine LTX/PLUS reagent (Thermo Fisher). Two days after transfection, cells were incubated with 10 μM sorafenib for a short period of 4 hours, fresh culture medium without sorafenib was then used to replace drug-containing medium and cell viability was determined by the MTT test 68 h later. The results were compared with those obtained in non-transfected HEL cells.

### RT-qPCR

Total RNA extraction was carried out using the illustra RNAspin Mini RNA Isolation Kit (GE Healthcare Life Sciences, Barcelona, Spain), RNA concentration was determined using a Nanodrop Spectrophotometer (ThermoScientific) and retrotranscription was performed using a high-capacity cDNA reverse transcription kit (Applied Biosystems, Madrid). Real-time quantitative PCR (qPCR) was performed using AmpliTaq Gold polymerase (Applied Biosystems) in an ABI Prism 7300 Sequence Detection System (Applied Biosystems) with the following thermal conditions: 1 cycle of 95°C for 10 min and 40 cycles of 95°C for 15 s and 60°C for 60 s. The primer oligonucleotide sequences to carry out qPCR are shown in Supplementary Table 1. At the end of each reaction, a melting curve analysis was performed. The 2^−ΔΔCt^ method was applied to analyze the relative expression of each gene and glyceraldehyde 3-phosphate dehydrogenase (GAPDH), which was used as the reference gene. For each cell line, the analysis was performed in cDNA obtained from two independent cell cultures and quantification was performed in duplicates.

### Functional studies

Carboxyfluorescein, rhodamine-123 and BODIPY-prazosin or mitoxantrone efflux assays were carried out to evaluate the functional activity of MRP, MDR1 and BCRP pumps, respectively, by flow cytometry. DHE uptake was determined to evaluate OCT1 functionality. Briefly, cells were incubated in 100 μl of uptake medium (96 mM NaCl, 5.3 mM KCl, 1.1 mM KH_2_PO_4_, 0.8 mM MgSO_4_, 1.8 mM CaCl_2_, 11 mM glucose, and 50 mM HEPES, pH 7.40) containing 1 μM carboxyfluorescein, rhodamine-123 or BODIPY-prazosin or 25 μM mitoxantrone at 37°C for the indicated time periods. In some experiments, probenecid, a typical MRP inhibitor, verapamil, a typical MDR1 inhibitor, or fumitremorgin C, a typical BCRP inhibitor, or other more specific MRP inhibitors (Supplementary Table 2) were added together with the appropriate substrate. Then, 900 μl of ice-cold uptake medium was added to stop the transport process, and intracellular fluorescence was determined immediately by flow cytometry. Uptake studies were performed, as previously reported [28], using DHE as OCT1 substrate and 2-chloro-MP as inhibitor, after selecting the latter as the best inhibitor. Chloro MP-sensitive uptake of DHE uptake was also measured in HEL cells transiently transfected with wild-type OCT1 or a mutated form of OCT1. HepG2 cells stably expressing OCT1 were used as a positive control. Uptake and efflux times were chosen based on previous studies of time-course that are routinely performed to fix the conditions.

### Statistical analysis

Data are presented as means±SEM. Paired or unpaired Student *t*-tests, as appropriate, or the Bonferroni method of multiple comparison test, were used to calculate the statistical significance of differences among groups.

## SUPPLEMENTARY MATERIALS TABLES



## Abbreviations

AML: acute myeloid leukemia
BCRP: breast cancer resistance protein
FLT3: Fms-like tyrosine kinase 3
GAPDH: glyceraldehyde-3-phosphate dehydrogenase
MDR1: multidrug resistance protein
MRP: multidrug resistance-associated protein
OATP: organic anion-transporting polypeptide
OCT: organic cation transporter
TKR: tyrosine kinase receptor
