# Supplementary material for: Role of drug transporters in the sensitivity of acute myeloid leukemia to sorafenib
Source: Oncotarget. 2018 Jun 19;9(47):28474–85. doi: 10.18632/oncotarget.25494 (PMC6033373; doi:10.18632/oncotarget.25494)
Supplement: Supplementary file 1 [file oncotarget-09-28474-s001.pdf]

Role of drug transporters in the sensitivity of acute myeloid leukemia to sorafenib

SUPPLEMENTARY MATERIALS

Supplementary Table 1: Gene-specific oligonucleotide sequences for primers used in real-time RT-QPCR

Protein/Gene	Forward primer (5'-3')	Reverse primer (5'-3')	Product size (bp)	Accession number
OCT1 (*SLC22A1*)	TGCAGACAGGTTTGGCCGT	GCCCGAGCCAACAAATTCTGTGAT	187	NM_003057
OCT2 (*SLC22A2*)	CCCTTCAGCGCCTGAGACTT	TCACCTGCAAGGCCCATG	177	NM_003058
OCT3 (*SLC22A3*)	CATCGTCAGCGAGTTTGAC CTTGT	GTAAATGACGATCCTGCCAT ACCTGTCT	139	NM_021977
MDR1 (*ABCB1*)	GCGCGAGGTCGGAATGGAT	CCATGGATGATGGCAGCCAAAGTT	198	NM_000927
BCRP (*ABCG2*)	CCCAGGCCTCTATAGCTCA GATCATT	CACGGCTGAAACACTGCTGAAACA	161	NM_004827
MRP2 (*ABCC2*)	TGAAGAGGAAGCCACAGT CCATGA	TTCAGATGCCTGCCATTGGACCTA	171	NM_000392
MRP3 (*ABCC3*)	CCAAGTTCTGGGACTCCAA CCTG	ATGATGTAGCCACGACAATGGTGC	160	NM_003786
MRP4 (*ABCC4*)	TGCAAGGGTTCTGGGAT AAAGA	CTTTGGCACTTTCCTCAATTAACG	141	NM_005845
MRP5 (*ABCC5*)	GTTCAGGAGAACTCGA CCGTTGG	TTTGGAAGTAGTCCGGATGGGCTT	178	NM_005688
FLT3 (*FLT3*)	TCAAGATCTGCCTGTGATC AAGTGTGT	CCGGGGATTCTGATACCATGGGATA	101	NM_004119
KIT (*KIT*)	CGTCCAGACAGGCTCT TCTCAA	GACAAAGCCCGGATCAGTGCATAA	136	NM_001093772
GAPDH (*GAPDH*)	TGAGCCCGCAGCCTCC	TACGACCAAATCCGTTGACTCC	138	NM_002046

Detection of the amplification products was carried out using SYBR Green I. The mRNA abundance of target genes in each cell line was normalized on the basis of mRNA GAPDH abundance.

Supplementary Table 2: Selected substrates and inhibitors used in this study

Protein/s	Fluorescent substrates
MDR1	Rhodamine 123
MRP3/4/5	Carboxyfluorescein*
BCRP	BODIPY-prazosin
BCRP	Mitoxantrone
OCT1	Dihydroethidium (DHE)
**Protein/s**	**Inhibitors**
MDR1	Verapamil
MRP3	p-acetamidophenyl β-D-glucuronide (APAP-gluc)
MRP3/4	Taurochenodeoxycholic acid (TCDCA)
MRP4/5	Guanosine 3′,5′-cyclic monophosphate (cGMP)
BCRP	The inhibitor of BCRP is fumitremorgin C (FTC)
OCT1	The inhibitor of OCT1 is 2-chloro-1-methylpyridinium iodide (chloro-MP)

BCRP, breast cancer resistance protein; MDR1, multidrug resistance 1; MRP, multidrug resistance-associated protein; OCT1, organic cation transporter 1.

(*) Carboxyfluorescein was added to the uptake medium as a non-fluorescent ester.
